# Supplementary material for: Investigating the Mitoprotective Effects of S1P Receptor Modulators Ex Vivo Using a Novel Semi-Automated Live Imaging Set-Up
Source: Int J Mol Sci. 2023 Dec 23;25(1):261. doi: 10.3390/ijms25010261 (PMC10778583; doi:10.3390/ijms25010261)
Supplement: Supplementary file 1 [file ijms-25-00261-s001.zip › ijms-2727085-supplementary.pdf]

```
if (nImages == 0)
	open();
title = getTitle();
//Properties and movement correction
run("Properties...", "channels=1 slices=1 frames=31 unit=um pixel_width=0.34 pixel_height=0.34 voxel_depth=0.34 frame=[4sec]");
setOption("ScaleConversions", true);
run("StackReg ", "transformation=[Rigid Body]");
// Enhance Contrast
run("Enhance Contrast", "saturated=0.35");

//Movement tracking with TrackMate plugin --> MANUALLY
//run("TrackMate"); //Values must be set manually
waitForUser("Motility data saved and continue with morphology analysis"); //Save motility data

//Morphology analysis with ImageJ
getLocationAndSize(x, y, width, height);
run("Set Measurements...", "area perimeter shape feret's redirect=None decimal=3");
run("Duplicate...", "title=Mask ignore range=1 use");
run("Remove Overlay");
resetMinAndMax();
run("8-bit");
setLocation(x, y);
run("Duplicate...", "title=Vis ignore range=1 use");
setLocation(x + width, y);
run("Green");
selectImage("Mask");
setAutoThreshold("Default dark");
call("ij.plugin.frame.ThresholdAdjuster.setMode", "B&W");
run("Threshold...");
waitForUser("Analyze");
close("Threshold");
close("Vis");
setBatchMode(1);
run("Analyze Particles...", "size=0.20-5.00 show=[Bare Outlines] display clear summarize");
selectWindow("Drawing of Mask");
run("Invert");
run("Red");
selectWindow("Mask");
run("Green");
run("RGB Color");
imageCalculator("Transparent-zero", "Mask","Drawing of Mask");
close("Drawing of Mask");
setBatchMode(0);
run("Tile");
exit
```

Supplementary Figure S1. In-house developed ImageJ Macro for semi-automated analysis.

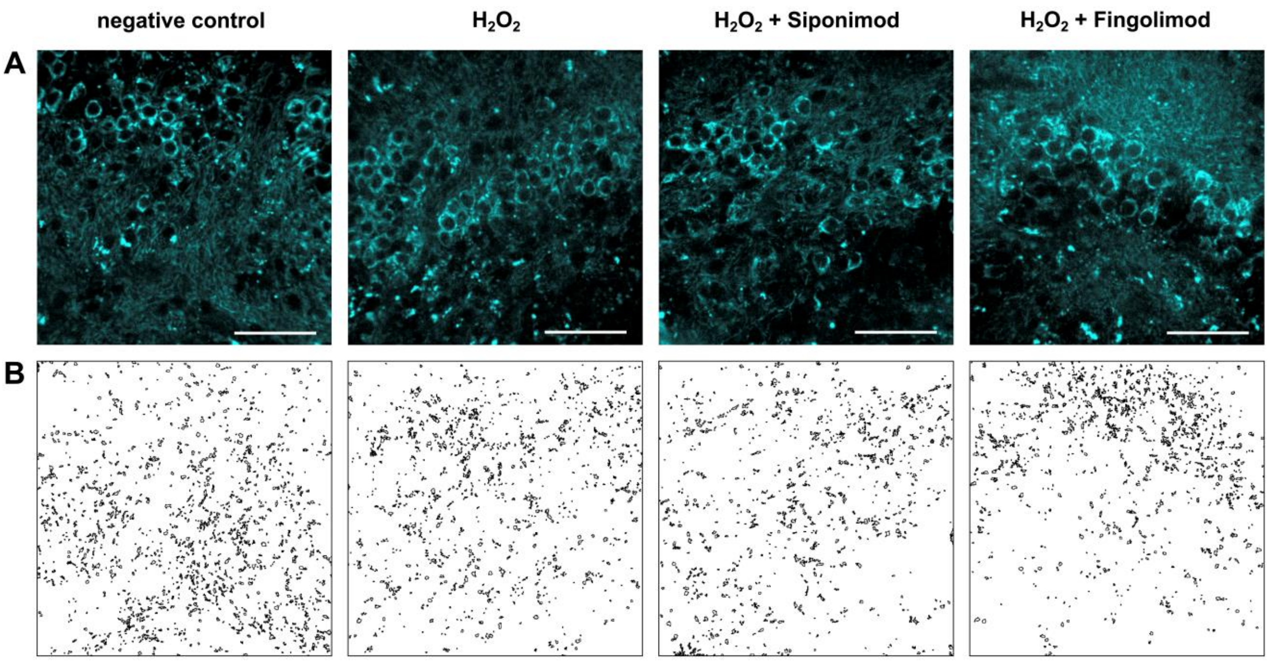

Supplementary Figure S2. Pre- and post-segmentation images for morphological analysis of mitochondrial area, length, and aspect ratio. **A** Exemplary DG fluorescence images of all four treatment conditions in which morphological analysis was performed (first image of stack; 40x Apo Water Objective). Scale bars: 50 µm. **B** ImageJ masks used for particle analysis showing bare outlines of assessed mitochondria for the respective ROIs shown in A (only particles with a size of 0.20-5.00 μm^2 were considered). The length of mitochondria decreased upon oxidative stress and significantly increased again upon treatment of H_2O_2 + siponimod, showing a similar trend for H_2O_2 + fingolimod (mean mitochondrial length in exemplary ROIs: neg. control=1.499 µm; H_2O_2=1.387 µm; H_2O_2 + siponimod=1.500 µm; H_2O_2 + fingolimod=1.428 µm).

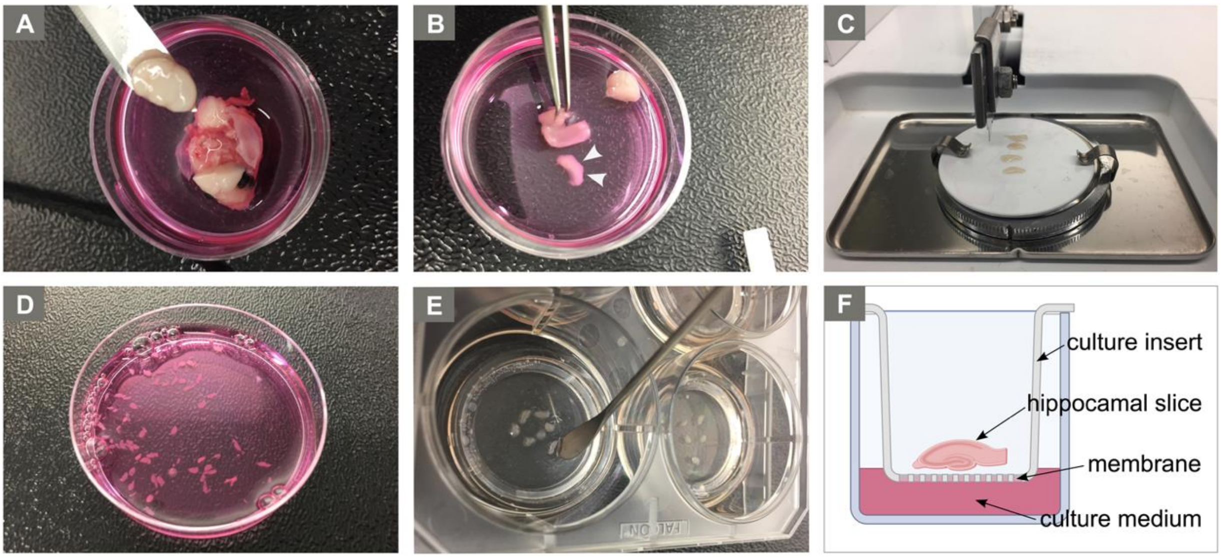

Supplementary Figure S3. Generation of organotypic hippocampal slice cultures using a McIlwain Tissue Chopper**.** **A** Coronal separation of brain into three parts with middle part containing the hippocampus. **B** Isolation of right hippocampus (indicated by white arrowheads). **C** Hippocampi arranged on the cutting disc of the Tissue Chopper prior to slicing. **D** 350 µm thick hippocampal slices in sterile culture medium. **E** Transfer of slices to membranes of culture inserts. **F** Schematic illustration of the incubation setup showing a hippocampal slice on top of the membrane of a culture insert in a 6-well plate. Supply with culture medium is ensured via the permeable membrane (pore size 0.4 µm).
